# Clinically relevant burden of delayed gastric emptying after left pancreatectomy and its predictors

**DOI:** 10.1007/s13304-026-02593-y

**Published:** 2026-03-24

**Authors:** Jingcheng Zhang, Carsten Jäger, Alper Doğruöz, Helmut Friess, Ihsan Ekin Demir, Florian Scheufele

**Affiliations:** Department of Surgery, Klinikum Rechts Der Isar, School of Medicine and Health, Technical University of Munich, Ismaninger Straße 22, 81675 Munich, Bavaria Germany

**Keywords:** Delayed gastric emptying, Left pancreatectomy, Pancreatic ductal adenocarcinoma, Portal vein, Superior mesenteric vein

## Abstract

Few studies have examined delayed gastric emptying (DGE) following left pancreatectomy (LP). This study aimed to assess the incidence and impact and identify predictive pre/intra-operative predictors. We conducted a retrospective, single-centre cohort including all adult patients who underwent LP from 2017 to 2024. Variables were analyzed using univariate and multivariable analysis. Among 213 LP patients, 34 (16.0%) developed DGE (grade A 11.7%, B 3.3%, C 0.9%). DGE was associated with longer hospitalization [30.50 (20.75–46.50) vs 14.00 (11.00–20.00) days, *P* < 0.001], higher major complications (67.6% vs 29.6%, *P* < 0.001), and more ICU stays (29.4% vs 13.4%, *P* = 0.025); even isolated grade A DGE (no other complications) prolonged length of stay than in patients without any complications [20.00 (15.00–29.50) vs 12.00 (10.00–15.00) days, *P* = 0.021]. DGE was linked to clinically relevant POPF (52.9% vs 29.6%; *P* = 0.010) and intra-abdominal abscess with invasive therapy (20.6% vs 6.1%; *P* = 0.015). In multivariable analysis, portal (PV)/superior mesenteric vein (SMV) resection (OR 4.525, *P* = 0.017) and pancreatic ductal adenocarcinoma (PDAC) histology (OR 3.121, *P* = 0.024) were independent predictors of DGE. DGE is a frequent and under-estimated complication after LP, prolonging postoperative hospitalization; notably, even grade A DGE was linked to longer length of stay. PDAC histology and PV/SMV resection were independent risk factors for DGE. Identifying these predictors enables prediction of DGE and supports targeted prevention and perioperative management.

**Supplementary Information:**

The online version contains supplementary material available at 10.1007/s13304-026-02593-y.

## Introduction

Left pancreatectomy (LP) is a common treatment for benign and malignant tumors, chronic inflammation, and trauma in the tail or body of the pancreas [1, 2]. Compared with pancreaticoduodenectomy (PD), LP is thought to have lower mortality and morbidity and thus does not receive much attention [3, 4]. However, despite advancements in surgical techniques, such as the incorporation of laparoscopy and robotics, the complications following LP remain prevalent [5].

Delayed gastric emptying (DGE) represents the most frequent complication after pancreaticoduodenectomy (PD), marked by the incapacity to resume a standard diet during the initial postoperative week [6]. DGE often necessitates prolonged nasogastric intubation for the affected patient, markedly prolongs a patient’s postoperative hospitalization, and imposes a financial burden [7, 8]. However, only a proportion of patients of DGE patients undergo formal testing and diagnosis due to its low life-threatening risk, which underscores a pervasive lack of awareness among surgeons regarding DGE following pancreatectomy [9]. Particularly, it is still under-investigated in the current exploration of DGE after LP, which could well aid in the early identification of DGE for better prevention and treatment [10]. This study aimed to assess the incidence of DGE after LP and its impact on short-term outcomes and to identify predictive risk factors.

## Materials and methods

### Study population

This retrospective single-centre study was reviewed by the Ethics Committee of the Technical University of Munich (Munich, Germany), which raised no objections to its conduct (reference 66/19 S; date 12 February 2019). The study was conducted in accordance with the Declaration of Helsinki. Written informed consent for inclusion in the institutional pancreatic surgery database and for scientific analysis and publication of de-identified data was obtained from all participants prior to database entry. We consecutively included all adult patients who underwent LP between 2017 and 2024, with exclusion criteria of incomplete baseline information. All surgeries were performed at a pancreatic center with an annual pancreatic surgery volume exceeding 100 cases, and all surgeons were specialized pancreatic surgeons who had undergone professional training.

### Data collection

Variables including preoperative demographics, comorbidities, laboratory results, intraoperative procedure and time, pathologic findings, postoperative complications, and short-term outcomes were obtained from a prospective database. To minimize misclassification, we conducted a comprehensive secondary review of each patient’s DGE status. Certain variables were excluded due to a high proportion of missing data, while others were omitted due to a low number of events, which could compromise statistical validity.

The definition and grading of DGE, postoperative pancreatic fistula (POPF), and post-pancreatectomy hemorrhage (PPH) in this study were based on the criteria established by the International Study Group of Pancreatic Surgery (ISGPS), in which DGE was categorized into grade A, B, and C based on nasogastric tube duration, tolerance to solid food, and symptoms of vomiting/gastric distension [8, 11, 12]. The age cut-off of ≥ 80 years was selected based on demographic trends and prior research [13]. A BMI threshold of 30 kg/m^2^ was chosen based on the World Health Organization (WHO) international classification, which defines BMI ≥ 30 kg/m^2^ as obesity. Preoperative weight loss was determined based on patient-reported history and documented in the clinical record system at the time of hospital admission.

### LP procedure

Standard LP was performed using either an open or minimally invasive/laparoscopic approach. In cases of malignancy, a radical R0 resection was conducted, including lymphadenectomy and splenectomy. The pancreas was typically divided at the level of the venous confluence, and intraoperative frozen section analysis was used to confirm tumor-free resection margins. If margins were positive, the extent of resection was adjusted accordingly. For open LP, the pancreatic parenchyma was divided using a scalpel, while for minimally invasive LP, division of the pancreas was performed with a stapler. Surgical passive drains were routinely placed at the pancreatic stump.

### Perioperative management and DGE treatment

Per institutional standard, a nasogastric tube is placed perioperatively in all patients undergoing LP and is typically removed on postoperative day 1. Oral intake is then advanced in a stepwise manner. In patients with intolerance to oral intake or recurrent nausea/vomiting suggestive of postoperative gastric dysfunction, oral intake is reduced (often to liquids) and prokinetic therapy is initiated, most commonly metoclopramide 10 mg three times daily; if clinically indicated, erythromycin 250 mg intravenously three times daily is added. Supportive intravenous nutrition is provided according to clinical judgment when adequate oral intake cannot be maintained. Postoperative abdominal CT imaging is not performed routinely; it is obtained on demand when intra-abdominal complications are suspected, including changes in drain output, significant abdominal pain, or other signs of clinical deterioration.

### Statistical analysis

Categorical variables were represented as frequencies with percentages and compared with the chi-square or Fisher’s exact test as appropriate. The distribution of continuous variables was examined using the Shapiro–Wilk test. Parametric continuous variables were reported with mean and standard deviations (mean ± SD), and the t-test was used for comparative analyses. Non-parametric continuous variables were shown with the median and interquartile range and evaluated by the Mann–Whitney U test. Candidate predictors were prespecified as pre- and intraoperative variables. Postoperative complications were excluded to preserve temporal ordering and avoid collider bias. To address missing data, multiple imputation was applied for variables with moderate missing rates (less than 40%). A 2-sided *P*-value less than 0.05 was defined as statistically significant. All statistical analyses were conducted on SPSS 27.0 and GraphPad Prism 9.0.0.

## Results

### The incidence of DGE after LP

A total of 213 patients who underwent LP surgery from February 2017 to October 2024 were enrolled. Of these, 158 patients underwent standard LP, while remaining 55 patients (25.8%) were operated “extended” LP involving multivisceral resection, including the resection of the adrenal gland (n = 19), small bowel (n = 2), portal vein (PV)/superior mesenteric vein (SMV) (n = 8), colon (n = 6), adrenal gland & PV/SMV (n = 5), adrenal gland & liver (n = 5), adrenal gland & colon (n = 5), PV/SMV & small bowel (n = 1), adrenal gland & colon & PV/SMV (n = 1), adrenal gland & celiac trunk & PV/SMV (n = 1), colon & celiac trunk & PV/SMV (n = 1) and liver & celiac trunk & PV/SMV (n = 1). Postoperatively, 34 patients (16.0%) developed DGE, which was classified as grade A (n = 25, 11.7%), grade B (n = 7, 3.3%), or grade C (n = 2, 0.9%), in contrast to the remaining 179 patients (84.0%) without DGE. The incidence of clinically relevant DGE (CR-DGE), combined with grade B and C, was 4.2%. In the standard (non-extended) LP subgroup (n = 158), DGE occurred in 18 patients (11.4%), including grade A/B/C DGE in 13/4/1 patients (8.2%/2.5%/0.6%), respectively.

### Complications and short-term outcomes

**Fig. 1:**
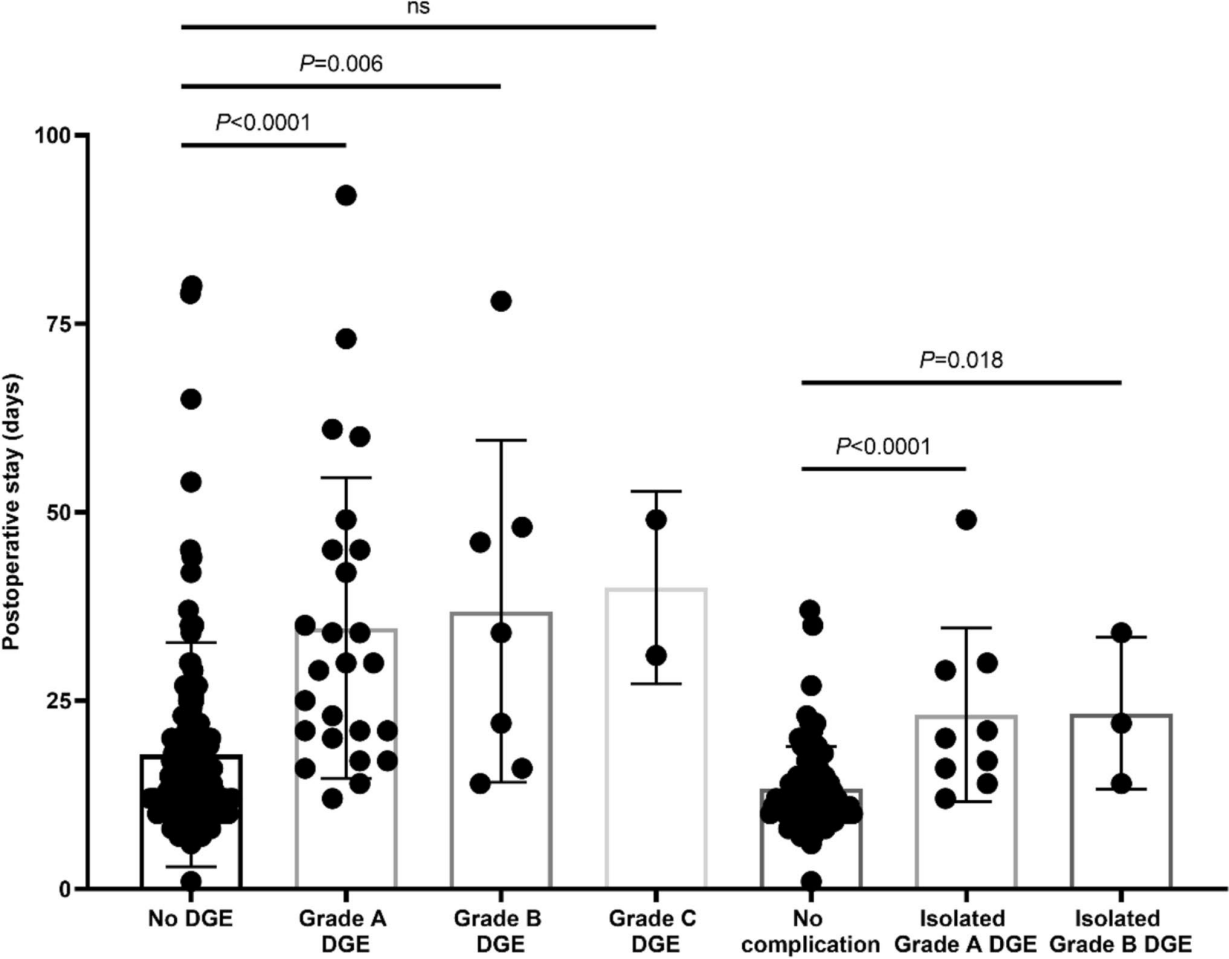
The length of hospitalization after LP in patients with different DGE classifications. *P* values are two-sided from the Mann–Whitney U test. Isolated grade A/B DGE, the DGE A/B patients without any other complications. LP, left pancreatectomy; DGE, delayed gastric emptying

Details concerning other postoperative complications and short-term outcomes in both groups are shown in Table 1. Compared with the patients without DGE, DGE patients had a significantly longer postoperative stay [30.50 (20.75–46.50) vs 14.00 (11.00–20.00) days, *P* < 0.001] and higher rate of major complications (Clavien-Dindo classification ≥ III, 67.6% vs 29.6%, *P* < 0.001). Specifically, the postoperative length of stay was markedly increased in grade A DGE [30.00 (20.50–45.00) days, *P* < 0.0001] compared with non-DGE [14.00 (11.00–20.00) days], and likewise in grade B DGE [34.00 (16.00–48.00) days, *P* = 0.024]. To minimize confounding by other complications, we also compared the length of stay between patients with isolated DGE A/B and those without any complications. Patients with isolated DGE A (n = 9) and DGE B (n = 3) had significantly longer hospitalizations than the no-complication group (n = 87) [20.00 (15.00–29.50) vs 12.00 (10.00–15.00) days, *P* = 0.021; 22.00 (14.00–34.00) vs 12.00 (10.00–15.00) days, *P* = 0.039] (Fig. 1). DGE was associated with higher rates of clinically relevant POPF (52.9% vs 29.6%; *P* = 0.010) and intra-abdominal abscess requiring invasive therapy (20.6% vs 6.1%; *P* = 0.015), and ICU stay was more frequent in the DGE group (29.4% vs 13.4%; *P* = 0.025). Other postoperative events, including PPH, postoperative wound infection, other surgical complications, other non-surgical complications, inpatient readmission, and 30-day/90-day mortality did not differ between groups.

**Table 1:** Postoperative characteristics of LP patients with and without DGE

	Non-DGE	DGE	*P*
No. of patients	179	34	
Clinically relevant POPF	53 (29.6)	18 (52.9)	0.010
Clinically relevant PPH	14 (7.8)	5 (14.7)	0.336
Wound infection	14 (7.8)	2(5.9)	0.969
Intra-abdominal abscess with invasive therapy	11 (6.1)	7(20.6)	0.015
Other surgical complications	18 (10.1)	4 (11.8)	1.000
Other non-surgical complications	25 (14.0)	5 (14.7)	1.000
Clavien-Dindo classification ≥ III	53 (29.6)	23 (67.6)	0.000
Psycho-oncological care needed	33 (18.4)	7 (20.6)	0.100
Stay in ICU	24 (13.4)	10 (29.4)	0.025
Inpatient readmission	25 (14.0)	5 (14.7)	1.000
30-day mortality	1 (0.6)	0	1.000
90-day mortality	3 (1.7)	0	1.000
Postoperative stay (day)	14.00 (11.00–20.00)	30.50 (20.75–46.50)	< 0.001

LP, left pancreatectomy; DGE, delayed gastric emptying; POPF, postoperative pancreatic fistula; PPH, post-pancreatectomy hemorrhage; ICU, intensive care unit

### Demographics and preoperative clinical characteristics

**Fig. 2:**
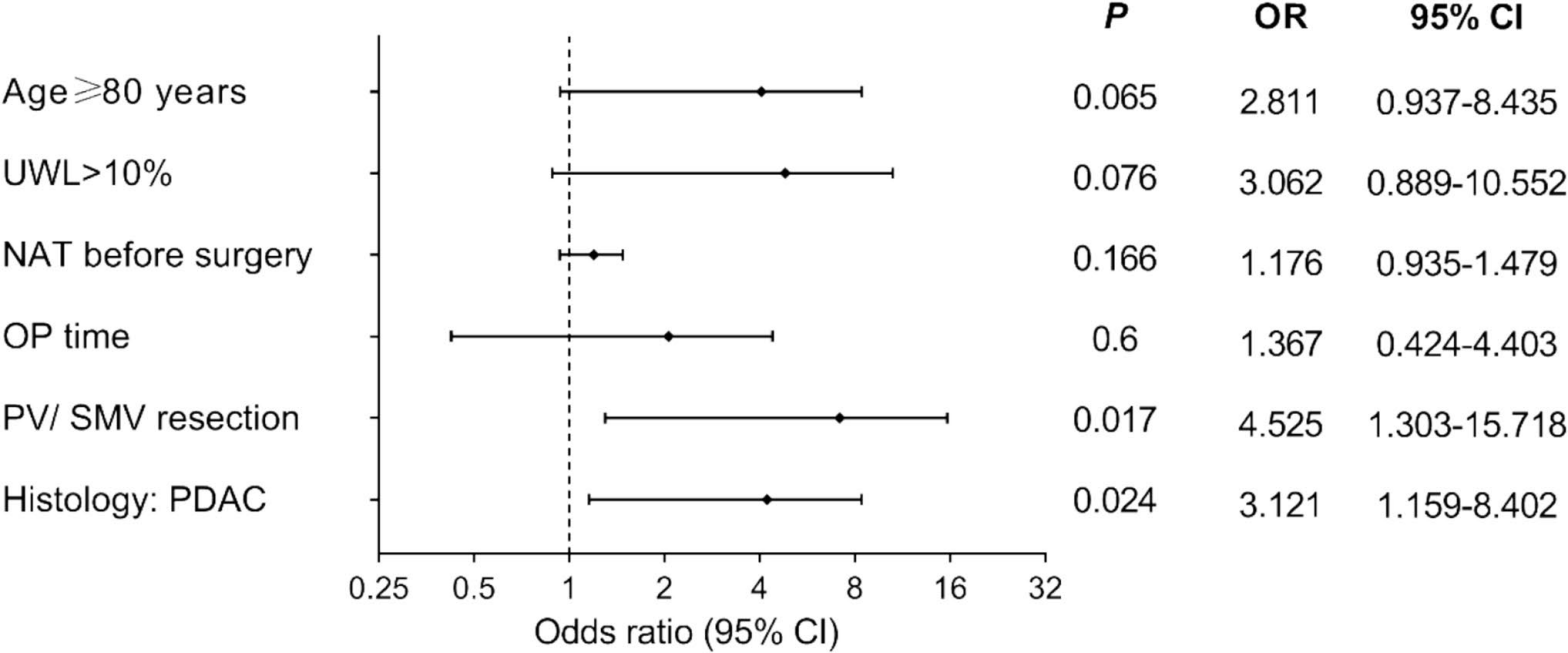
Forest plot of multivariable analysis of independent risk factors for DGE after LP Forest plot of adjusted odds ratios (ORs) with 95% confidence intervals (CI) from a multivariable logistic regression including pre- and intra-operative variables. DGE, delayed gastric emptying; LP, left pancreatectomy; UWL, unintentional weight loss; OP, operation; PV, portal vein; SMV, superior mesenteric vein; PDAC, pancreatic ductal adenocarcinoma

Comparisons of patient demographics and preoperative clinical characteristics for those patients having DGE versus those who did not are shown in Tables 2 and S1. Two preoperative factors were associated with DGE: age ≥ 80 years (20.6% vs 8.4%; *P* = 0.032) and unintentional weight loss (UWL) > 10% within 6 months before surgery (17.6% vs 6.1%; *P* = 0.023). In addition, neoadjuvant therapy (NAT) within 3 months before surgery was more frequent in the DGE group (29.4% vs 13.4%; *P* = 0.020). Other characteristics, including gender, BMI, ASA, elective surgery, smoking, alcohol abuse within 1 year before surgery, nausea or vomiting, pancreatitis, diabetes, severe COPD, high blood pressure medication, anticoagulant therapy, biliary stent, perioperative antibiotics, carcinoembryonic antigen (CEA), and carbohydrate antigen 19–9 (CA19-9), were not associated with DGE after LP (Fig. 2).

**Table 2:** Demographics, preoperative, and intraoperative characteristics of LP patients with and without DGE

	Non-DGE	DGE	*P*
Male/female gender, %	50.3/49.7	35.3/64.7	0.109
Age ≥ 80 years	15 (8.4)	7 (20.6)	0.032
BMI ≥ 30	26 (14.5)	2 (5.9)	0.276
ASA III + IV	68 (38.0)	16 (47.1)	0.321
UWL > 10% within 6 months before surgery	11 (6.1)	6 (17.6)	0.023
Symptoms: pain	47 (26.3)	12 (35.0)	0.280
Symptom: nausea or vomiting	6 (3.4)	2 (5.9)	0.826
Diabetes	45 (25.1)	8 (23.5)	0.842
Smoking	42 (23.5)	9 (26.5)	0.706
Alcohol abuse within 1 year before surgery	22 (12.3)	2 (5.9)	0.431
NAT within 3 months before surgery	24 (13.4)	10 (29.4)	0.020
Preoperative anticoagulant therapy	33 (18.4)	4 (11.8)	0.347
Perioperative antibiotics	10 (5.6)	4 (11.8)	0.339
Perioperative biliary stent placement	6 (3.4)	1 (2.9)	1.000
Spleen preservation	24 (13.4)	9 (26.5)	0.641
Laparoscopic surgery	37 (20.7)	3 (8.8)	0.105
Time of operation	235.00 (186.00–301.00)	283.00(230.00–378.00)	0.025
Pancreas texture: soft	111(62.0)	25 (73.5)	0.331
Pancreas duct diameter < 3 mm	120 (67.0)	21 (61.8)	0.298
PV/ SMV resection	9 (5.0)	9 (26.5)	< 0.001
Infusion of erythrocytes concentrates	17 (9.5)	4 (11.8%)	0.926

LP, left pancreatectomy; DGE, delayed gastric emptying; BMI, body mass index; ASA, American society of anesthesiologists; UWL, unintentional weight loss; NAT, neoadjuvant therapy; PV, portal vein; SMV, superior mesenteric vein; PDAC, pancreatic ductal adenocarcinoma

### Intraoperative characteristics

As shown in Tables 2 and S1, patients with DGE had a longer operative time [283.00 (230.00–378.00) vs 235.00 (186.00–301.00) min; *P* = 0.025] and more often underwent PV/SMV resection (26.5% vs 5.0%; *P* < 0.001). DGE patients were less likely to undergo laparoscopic techniques compared with non-DGE patients (8.8% vs 20.7%) but did not reach significance (*P* = 0.105). Other operative factors, including spleen, colon resection, small bowel resection, liver resection, and infusion of erythrocyte concentrates did not differ between the two groups.

### Histologic characteristics

Analysis of the distribution of primary diseases revealed a higher proportion of pancreatic ductal adenocarcinoma (PDAC) among patients with DGE compared to those without DGE (79.4% vs 43.6%, *P* = 0.005). Further analysis of pathological factors in 105 PDAC patients revealed that T stage, the number of examined/infiltrated lymph nodes N stage, perineural invasion (Pn), lymphatic vessels invasion (L), and vein invasion (V) were similar between the two groups.

### Independent pre- and intra-operative risk factors for DGE after LP

In the multivariable model, PV/SMV resection remained independently associated with DGE [R 4.525, 95% CI 1.303–15.718;* P* = 0.017], as did PDAC histology [OR 3.121, 95% CI 1.159–8.402; *P* = 0.024]. Age ≥ 80 years [OR 2.811, 95% CI 0.937–8.435;* P* = 0.065] and UWL > 10% within 6 months [OR 3.062, 95% CI 0.889–10.552; *P* = 0.076] showed positive trends but did not reach statistical significance. Operative time and NAT within 3 months before surgery were not independently related to DGE.

## Discussion

In the present study, DGE was retrospectively examined in a prospective database including 213 patients who underwent LP from a single institution, covering incidence, short-term outcomes, and predictive risk factors. The overall DGE rate after LP was 16.0%, which is within the range of 4.2–24.0% found in previous studies [5, 14–18]. Compared with the pooled 5.0% DGE incidence after LP in a recent meta-analysis involving 35,248 patients, our rate after LP was higher[19]. This difference likely reflects a more complex case mix in our cohort, including 25.8% with extended multivisceral resection, and strict outcome adjudication with secondary review of all cases.

There is a consensus that DGE after pancreatic surgery can lead to prolonged postoperative hospitalization, resulting in negative patient experience as well as increased financial burden [20]. Our study corroborates this, showing that patients with DGE experienced substantially longer hospital stays together with higher rates of major complications and postoperative ICU admission. Notably, even grade A DGE, comprising up to 73.5% of all DGE cases in this study, remained associated with prolonged hospitalization and this association persisted between isolated DGE A/B patients and those without any complications. However, the underlying explanation is uncertain: given the retrospective design and the time-dependent nature of DGE diagnosis, we cannot conclusively determine whether grade A DGE directly drives delayed discharge or whether it also reflects slower global recovery, greater frailty, higher surgical complexity, or conservative institutional feeding and discharge practices. In a German-wide multicenter study including 1688 LP patients, grade A DGE accounted for 61.3% of all DGE cases [17], and also reached 67.7% in a study including 311 patients [16]. Regrettably, neither study included a detailed analysis of grade A DGE. Many past studies may have overlooked grade A DGE, which merely demonstrated the prevalence of grade B/C DGE [21–23]. For POPF, the International Study Group of Pancreatic Surgery (ISGPS) updated the definition of pancreatic fistula in 2016, in which grade A POPF was renamed biochemical fistula, no longer considered to be a true pancreatic fistula, and not recognized as clinically important [12]. Per the 2007 ISGPS definition [8], grade A DGE is not linked to “major delay” in discharge; nevertheless, our results indicate that it is associated with a measurable postoperative burden, emphasizing the need to recognize and address even milder grade of DGE. This discrepancy may partly reflect procedure- and era-specific context: with modern laparoscopic/ Enhanced Recovery After Surgery (ERAS) LP and short baseline stays, even mild intake delays can prolong hospitalization, while after PD the longer baseline course and multiple co-determinants of length of stay may make grade A less discriminative for discharge timing. Future prospective studies incorporating granular perioperative process-of-care variables are needed to better disentangle biological effects from institutional practice patterns and to identify modifiable targets to reduce the burden of DGE after LP.

Moreover, in our cohort DGE was associated with clinically relevant POPF and with intra-abdominal abscess requiring intervention, a pattern consistent with previous evidence [16, 17]. In PD patients, POPF has even been postulated as the most significant factor in the development of DGE. Mechanistically, local sepsis and inflammatory cascades impair gastric motility and pyloroduodenal coordination, providing a biologically plausible pathway from POPF or abscess to DGE. Whereas for total pancreatectomy (TP), the incidence of DGE seems to be significantly higher than after LP or PD, at around 20.0%, which is clearly not due to POPF [24]. This observation highlights the intricate pathogenesis of DGE, and the dominant mechanisms may vary by surgical procedure. In the present analysis, we did not include POPF or abscess in multivariable modeling because the number of events was limited and the causal structure is complex, so we focused on pre- and intra-operative factors; future work with time-stamped complication data should clarify temporality and mediation.

This study identified PV/ SMV resection and PDAC histology as preoperative and intraoperative predictors for DGE after LP. In addition, both advanced age (≥ 80 years) and UWL > 10% within 6 months were strongly associated with DGE on univariable testing and showed concordant effects in adjusted models; their loss of significance likely reflects few events and wide confidence intervals. A previous study reported the relationship between age and DGE in LP [15], along with extensive research conducted in PD[25]. Weight loss as a diagnostic criterion for cancer cachexia leads to bad quality of life, tolerance and effectiveness of treatment, and increased mortality [26]. Past studies on UWL in PDAC have found that UWL > 10% was independently associated with worse survival and can serve as an early detection marker [27, 28]. UWL > 10% body weight emerges as a potentially independent risk factor for DGE after; however, confirmation in larger, preferably multicenter datasets is warranted. Following the supportive care practices of the Pancreatic Cancer Action Network [29], every patient with significant UWL, as needed, should receive pancreatic enzyme replacement therapy, nutritional supplements, pharmacologic interventions, and/ or be subjected to exercise, which may play a role in preventing DGE and merit prospective evaluation.

There were 18 patients with PV/SMV resection included in this study, with a significantly higher incidence of DGE (9/18, 50.0%), and all were diagnosed as PDAC. Given the high malignancy and invisible symptomatology of PDAC, up to one-third of all cases were considered borderline resectable or locally advanced due to the infiltration of surrounding major vessels [30]. Increasing evidence and experts’ consensus indicate that R0 resection achieved by pancreatectomy combined with PV/SMV resection and reconstruction results in satisfactory overall survival [30–32]. However, complications including DGE after PV/SMV resection are still controversial. A meta-analysis in 2016 evaluated the complications after PD with PV/SMV resection, which showed a higher rate of DGE compared with standard PD (12.6% vs 10.5%, *P* = 0.020) [33]. Another new meta-analysis showed that the incidence of DGE between different types of pancreatectomy (mixing PD, LP, and TP) with PV/SMV resection was similar to standard pancreatectomy [34]. Due to the closer anatomical relationship between the PV/SMV and the head of the pancreas rather than the body and tail of the pancreas, LP combined with PV/SMV is infrequent and relatively little studied. Recently, Dong X et al. first published research especially explored the short-term outcome of LP combining PV/ SMV resection, which, in accordance with the results of our study, showed a significantly higher rate of DGE than standard LP (9.8% vs. 1.2%, *P* = 0.042) [18]. Clearly, more research is still needed on LP combined with PV/ SMV resection surgery (Table 3).

**Table 3:** Pathological features of left pancreatectomy (LP) patients with and without delayed gastric emptying (DGE)

	Non-DGE	DGE	*P*
*Histological diagnosis*			0.005
PDAC	78 (43.6)	27 (79.4)	
Cystic tumor	42 (23.5)	2 (5.9)	
Neuroendocrine tumor	34 (19.0)	2 (5.9)	
Chronic pancreatitis	12 (6.7)	1 (2.9)	
Duodenal cancer	1 (0.7)	0 (0.0)	
Others	12 (6.7)	2 (5.9)	
Only for PDAC patients	78	27	
*T stage*			0.979
(y)T1-2	46 (59.0)	16 (59.3)	
(y)T3-4	32 (41.0)	11 (40.7)	
Lymph nodes examined	26.00 (19.00–34.00)	26.00 (16.75–36.75)	0.860
Lymph nodes infiltrated	1.00 (0.00–4.25)	2.00 (0.75–3.00)	0.723
*N stage*			0.100
(y) N0	31 (39.7)	6 (22.2)	
(y) N1-2	47 (60.3)	21 (77.8)	
Invasion into lymphatic vessels	37 (47.4)	12 (44.4)	0.964
Invasion into vein	20 (25.6)	7 (25.9)	0.977
Invasion into adjunct nerves	55 (70.5)	21 (77.8)	0.467
*R status*			0.552
R0	37 (47.4)	11 (40.7)	
R1	34 (43.6)	14 (51.9)	
Rx	7 (9.0)	2 (7.4)	

PDAC, pancreatic ductal adenocarcinoma

Additionally, PDAC histology emerged as an independent predictor of DGE in our LP cohort, which is comparable to the results of another study on LP [16, 19]. By contrast, several PD-centered studies have not found malignant pathology to be a consistent correlate of DGE, and have instead emphasized postoperative complications such as POPF as dominant drivers [25, 35]. Further analysis of various pathological characteristics in PDAC patients showed that DGE was not linked to the indicators of tumor aggressiveness including nodal status and other pathological indicators, indicating no measurable gradient by malignancy severity. The previously proposed explanation that higher DGE rates in PDAC might reflect denervation from lymphadenectomy is not supported by our data. Beyond surgical sequelae, PDAC-associated cachexia and systemic inflammation can plausibly impair gastric motility and perioperative resilience, which may heighten susceptibility to DGE and warrants prospective mechanistic study [26, 36].

In addition to the inherent biases of all retrospective analyses, including selection bias, recall bias, and information bias, this study has several other limitations. First, some preoperative laboratory data appeared to be missing. Regarding postoperative management variables, nasogastric tube management, prokinetic use, and nutritional support were largely standardized according to an institutional SOP, limiting between-patient variability. In contrast, granular data on opioid exposure and fluid balance were not systematically captured in our maintained database and therefore unavailable for analysis. Second, PV/SMV resection was performed only in PDAC patients in our cohort; therefore, some overlap between these predictors cannot be excluded, and their adjusted estimates should be interpreted with appropriate caution. Additionally, the relatively low events per variable (EPV) may have compromised the precision of the confidence intervals, as indicated by the wider intervals observed. Nevertheless, these data reflect real-world clinical practice at our center. Future studies with larger datasets and higher EPV thresholds are needed to substantiate these findings. Lastly, for patients with unintentional weight loss, specific percentages of weight loss were not available, restricting possible stratified analyses.

## Conclusions

In summary, DGE is a frequent and under-estimated complication after LP, prolonging postoperative hospitalization; notably, even grade A DGE was linked to longer length of stay. DGE was associated with CR-POPF and intra-abdominal abscess requiring invasive therapy. PDAC histology and PV/SMV resection were identified as independent risk factors for DGE following LP. Identifying these risk and association factors enables prediction of DGE and supports targeted prevention and perioperative management. Larger multi-centre cohorts with robust modelling and mediation analyses are needed to refine risk stratification and to test preventive strategies.

## Supplementary Information

Below is the link to the electronic supplementary material.

Supplementary file 1.

## Data Availability

The datasets generated and/or analyzed during the current study are available from the corresponding author on reasonable request.
