# Supplementary material for: Clinically relevant burden of delayed gastric emptying after left pancreatectomy and its predictors
Source: Updates Surg. 2026 Mar 24;78(3):1243–51. doi: 10.1007/s13304-026-02593-y (PMC13249675; doi:10.1007/s13304-026-02593-y)
Supplement: Supplementary file 1 [file 13304_2026_2593_MOESM1_ESM.docx]

**Clinically relevant burden of delayed gastric emptying after left pancreatectomy and its predictors**

Jingcheng Zhang, Carsten Jäger, Alper Doğruöz, Ihsan Ekin Demir, Helmut Friess, Florian Scheufele

^1^Department of Surgery, Klinikum rechts der Isar, School of Medicine and Health, Technical University of Munich, Munich, Germany

*Correspondence to*: Florian Scheufele, Department of Surgery, Klinikum rechts der Isar, School of Medicine and Health, Technical University of Munich, Ismaninger Straße 22, Munich, Bavaria 81675 Germany. ORCID ID: 0000-0002-7105-0895.

**Supplementary Materials - Index**

| **Supplementary Figures and Tables** |  |
| --- | --- |
| *Table S1* | *Page 1* |

**Table S1**. **Demographics, preoperative, and intraoperative characteristics of left pancreatectomy (LP) patients with and without delayed gastric emptying (DGE)**

|  | **Non-DGE** | **DGE** | ***P*** |
| --- | --- | --- | --- |
| Jaundice | 3 (1.7) | 0 (0.0) | 1.000 |
| *Comorbidities:* |  |  |  |
| Acute pancreatitis | 11 (6.1) | 1 (2.9) | 0.736 |
| Chronic pancreatitis | 14 (7.8) | 1 (2.9) | 0.513 |
| Coronary heart disease | 14 (7.8) | 1 (2.9) | 0.513 |
| Heart failure |  |  | 0.916 |
| NYHA I | 65 (36.3) | 13 (38.2) |  |
| NYHA II | 19 (10.6) | 4 (11.8) |  |
| NYHA III | 6 (3.4) | 0 (0.0) |  |
| Perioperative biliary stent placement | 6 (3.4) | 1 (2.9) | 1.000 |
| Multivisceral resection: colon | 9 (5.0) | 4 (11.8) | 0.265 |
| Multivisceral resection: small bowel | 2 (1.1) | 1 (2.9) | 0.408 |
| Multivisceral resection: liver | 4 (2.2) | 2 (5.9) | 0.540 |
| *Only for PDAC patients* |  |  |  |
| Preoperative CA 19-9 (U/ml) | 65.00 (21.00-246.00) | 167.00 (3.50-243.00) | 0.205 |
| CEA (ng/ml) | 2.40 (1.62-4.60) | 2.80 (1.87-5.34) | 0.511 |
| Colon resection | 9 (5.0) | 4 (11.8) | 0.265 |
| Small bowel resection | 2 (1.1) | 1 (2.9) | 0.408 |
| Liver resection | 4 (2.2) | 2 (5.9) | 0.540 |
| Celiac trunk resection |  |  |  |
| R1/R2 resection area: bile duct | 0 (0.0) | 1 (3.7) | 0.257 |
| R1/R2 resection area: duodenum | 1 (1.3) | 0 (0.0) | 1.000 |
| R1/R2 resection area: pancreas | 3 (3.8) | 1 (3.7) | 1.000 |
| R1/R2 resection area: ventral | 13 (16.7) | 5 (18.5) | 1.000 |
| R1/R2 resection area: medial | 1 (1.3) | 1 (3.7) | 0.450 |
| R1/R2 resection area: retroperitoneal | 15 (19.2) | 5 (18.5) | 0.935 |

COPD, chronic obstructive pulmonary disease; GGT, gamma-glutamyl transferase; AST, aspartate aminotransferase; CEA, carcinoembryonic antigen; CA 19-9, carbohydrate antigen 19-9.
